# Pandemic (H1N1) 2009 and Seasonal Influenza A (H1N1) Co-infection, New Zealand, 2009

**DOI:** 10.3201/eid1610.100116

**Published:** 2010-10

**Authors:** Matthew Peacey, Richard J. Hall, Stephanie Sonnberg, Mariette Ducatez, Shevaun Paine, Mackenzie Nicol, Jacqui C. Ralston, Don Bandaranayake, Virginia Hope, Richard J. Webby, Sue Huang

**Affiliations:** Author affiliations: The Institute of Environmental Science and Research, Upper Hutt, New Zealand (M. Peacey, R.J. Hall, S. Paine, M. Nicol, J.C. Ralston, D. Bandaranayake, V. Hope, S. Huang);; St. Jude Children’s Research Hospital, Memphis, Tennessee, USA (S. Sonnberg, M. Ducatez, R.J. Webby);; Australian National University, Canberra, Australian Capital Territory, Australia (S. Paine)

**Keywords:** Co-infection, dual infection, influenza, swine-origin influenza, pandemic A/H1N1 influenza 2009, viruses, New Zealand, dispatch

## Abstract

Co-infection with seasonal influenza A (H1N1) and pandemic (H1N1) 2009 could result in reassortant viruses that may acquire new characteristics of transmission, virulence, and oseltamivir susceptibility. Results from oseltamivir-sensitivity testing on viral culture suggested the possibility of co-infections with oseltamivir-resistant (seasonal A [H1N1]) and -susceptible (pandemic [H1N1] 2009) viruses.

Pandemic (H1N1) 2009 virus was first identified in mid-April 2009 (*1*), near the beginning of the Southern Hemisphere influenza season. The potential for reassortment of cocirculating seasonal influenza A viruses with pandemic (H1N1) 2009 virus within New Zealand generated considerable interest during the recent 2009 Southern Hemisphere influenza season (*2**,**3*). Of particular concern is the potential reassortment of neuraminidase gene segments leading to an oseltamivir-resistant pandemic strain.

Changes in the genome of pandemic (H1N1) 2009 virus by reassortment, recombination, or point mutation have the potential to alter the transmissibility, antigenicity, antiviral drug resistance, or virulence of the virus. Reassortment can occur when 2 viruses co-infect the same cell. The 8 influenza gene segments of each virus could then be exchanged, creating a reassortant virus. Pandemic (H1N1) 2009 is itself a reassortant virus containing gene segments of avian, human, and swine influenza virus origin (*4*). We report human co-infection with pandemic (H1N1) 2009 and seasonal influenza A (H1N1) viruses.

## The Study

Influenza viruses were identified through the New Zealand national influenza surveillance system as part of the World Health Organization global program for influenza surveillance previously reported (*2*). Pandemic (H1N1) 2009 virus dramatically increased demand for influenza subtyping (*2*), necessitating a change in the standard real-time reverse transcription–PCR (rRT-PCR) algorithm. Samples were first screened with singleplex universal influenza A and pandemic (H1N1) 2009 assays (*5**,**6*). If negative results were obtained for both of these tests, samples were then tested for influenza B. If samples were positive for universal influenza A but not for pandemic (H1N1) 2009 virus, they were subtyped for seasonal H1 and H3 by rRT-PCR. Samples positive for pandemic (H1N1) 2009 virus were not subsequently assayed for other influenza viruses during testing but were tested at the end of the Southern Hemisphere influenza season as part of this study.

By testing viral cultures of pandemic (H1N1) 2009 viruses for oseltamivir resistance, by fluorometric-inhibition assay (*7*), putative co-infections of resistant seasonal influenza A (H1N1) and susceptible pandemic (H1N1) 2009 were discovered; i.e., pandemic (H1N1) 2009 viruses initially appeared to be resistant to oseltamivir because of a co-infecting oseltamivir-resistant seasonal A (H1N1) virus in culture. Within New Zealand, all seasonal influenza A (H1N1) viruses tested during 2009 were oseltamivir resistant, and all pandemic (H1N1) 2009 viruses were susceptible (*3*).

After the discovery of co-infection in viral culture, 1,044 clinical samples that were positive for pandemic (H1N1) 2009 were screened by rRT-PCR for seasonal A (H1N1) virus. Eleven co-infections were identified. Two additional samples indicated co-infections when viral culture was screened by rRT-PCR but could not be confirmed because our laboratory did not receive the original clinical specimen.

Laboratory contamination of viral culture could account for the presence of both influenza subtypes in viral culture samples. Co-infection was confirmed by using World Health Organization–recommended specific singleplex rRT-PCRs (*5*) on each of the 11 original clinical specimens (Table). The specific rRT-PCRs each are specific for the gene segment encoding hemagglutinin; 1 assay is specific for pandemic (H1N1) 2009, the other for seasonal influenza A (H1N1). The 2 assays were run in parallel for each sample with appropriate controls, including specificity controls.

**Table:** Characteristics of 13 nonhospitalized patients co-infected with seasonal influenza A (H1N1) and pandemic (H1N1) 2009, New Zealand, June 2009*

Patient no.	rRT-PCR results, Ct				Patient characteristics					
	Sample type	Pandemic (H1N1) 2009	Seasonal A (H1N1)		Age, y/ sex	Ethnicity	Location	Date of onset	Received vaccination†	Received antiviral drug
1	Clinical	22.74	18.13		29/F	E	Manakau	14	No	Yes
2	Clinical	32.26	23.84		10/M	M	Rotorua	15	No	No
3	Clinical	23.97	17.87		13/M	M	Wellington	17	No	No
4	Clinical	26.04	32.96		18/F	E	Waikato	22	No	U
5	Clinical	22.67	34.68		22/F	ME	Wellington	22	Yes	No
6	Clinical	22.78	35.24		19/F	M	Bay of Plenty	23	Unknown	No
7	Clinical	18.03	33.58		36/M	E	Hamilton	23	No	Yes
8	Clinical	20.65	32.92		31/F	E	Hamilton	23	No	No
9	Clinical	23.69	31.5		24/F	M	Hamilton	24	No	No
10	Isolate	15.09	13.52		51/F	E	Hamilton	26	No	No
11	Clinical	30.77	22.76		21/F	U	Dunedin	28	No	Yes
12	Clinical	25.1	36.62		16/F	M	Rotorua	29	No	No
13	Isolate	29.93	12.54		17/F	E, M	Manukau	30	No	Yes

*rRT-PCR, real-time reverse transcription–PCR; Ct, cycle threshold; E, European; M, Maori; ME, Middle Eastern; U, unknown. †Patient history of receipt of seasonal influenza vaccination.

Within this small number of cases, 10 of the 13 patients were female, and 6 patients were of Maori descent. Each figure was higher than the expected 51% and 14.6% representation in the New Zealand population, respectively, but co-infections were too few to draw any conclusions based on these characteristics (Table).

A vaccine for pandemic (H1N1) 2009 was not available when these samples were collected (June–November 2009), and only 1 of the 13 patients had a history of seasonal influenza vaccination. None of the 13 case-patients had severe illness or were hospitalized.

Eight of the 13 case-patients came from the central North Island; the remainder came from Auckland (2), Wellington (2), and Otago (1). All of these regions had high influenza activity during the 2009 New Zealand influenza season (*2*).

For each case, details of initial and ongoing transmission were unclear. Two cases occurred in a husband and wife, who had onset of symptoms on the same day; the remaining cases are not thought to be linked. All cases were reported after pandemic (H1N1) 2009 had become widespread in the community; therefore, contact tracing had ceased within New Zealand.

Dates of illness onset for all case-patients occurred within a 16-day period (June 14–30). This period coincided with the short period when both seasonal A (H1N1) and pandemic (H1N1) 2009 viruses cocirculated at approximately equal levels in the community, before the pandemic virus became the predominant strain (*2*) (Figure).

**Figure:**
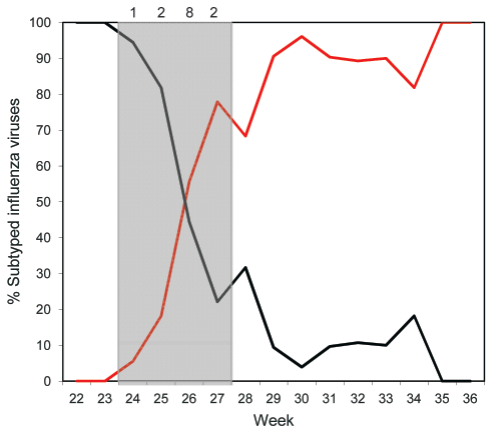
Co-infection during cocirculation of seasonal influenza A (H1N1) and pandemic (H1N1) 2009 viruses, New Zealand, 2009. Red line indicates pandemic (H1N1) 2009 viruses; black line indicates seasonal influenza A (H1N1) viruses. The gray shaded area indicates weeks in which the co-infections occurred; numbers above the graph indicate number of co-infections for that week: 1 co-infection in week 24, 2 in week 25, 8 in week 26, and 2 in week 27.

## Conclusions

Results from oseltamivir-sensitivity testing on viral culture suggested the possibility of co-infections in patients with both resistant (seasonal A [H1N1]) and susceptible (pandemic [H1N1] 2009) viruses. This test required both viruses to grow sufficiently in cell culture and grow to similar titers. Only by this approach was co-infection discovered and later investigated by use of more sensitive and highly specific rRT-PCRs.

Co-infections of different influenza viruses are rarely reported; reports focus solely on co-infections of influenza A and B, not of 2 influenza A subtypes (*8**–**11*). Two recent studies, 1 examining 2,273 clinical influenza samples with multiplex PCR methods found no influenza co-infections (*12**,**13*); another study estimated influenza co-infections to be as high as 3% (*14*). The rate of co-infection determined in this study was 1.1% (n = 1,044), which may underestimate the actual rate because not all tests used in this study (either biochemical or molecular) screened for co-infection of pandemic (H1N1) 2009 with other viruses, such as influenza A (H3N2) or influenza B.

Although influenza co-infections are rare, we have shown that they occurred during the first stage of a pandemic when seasonal strains cocirculated. This cocirculation poses a risk for further reassortment for the pandemic strain, which could result in a new pandemic strain. Of particular concern is the potential generation of an oseltamivir-resistant pandemic strain. The genesis of a harmful influenza reassortant warrants further investigation in animal models or in vitro systems. Further analysis of natural co-infections may help elucidate a role for the human host in influenza reassortment.
